# High‐mobility Group Protein 1/ Receptor for Advanced Glycation End Products/ Nuclear Factor‐κB Signalling Pathway Contributes to the Pathogenic Process of Striatal Neuron Impairment in the Rat Model of Parkinson's Disease

**DOI:** 10.1002/brb3.71133

**Published:** 2026-01-29

**Authors:** Yaofeng Zhu, Zean Du, Liping Sun, Ziyun Huang, Linju Jia, Tao Chen, Xuefeng Zheng, Wanlong Lei

**Affiliations:** ^1^ Institute of Medicine, College of Medicine Jishou University Jishou Hunan China; ^2^ Neuroscience Laboratory for Cognitive and Developmental Disorders, Department of Anatomy School of Medicine, Jinan University Guangzhou Guangdong China; ^3^ Department of Pathology, The Sixth Affiliated Hospital Sun Yat‐sen University Guangzhou Guangdong China; ^4^ Department of Anatomy, Zhongshan School of Medicine Sun Yat‐sen University Guangzhou Guangdong China

**Keywords:** dopamine depletion, neuronal damage, striatum, RAGE

## Abstract

**Background:**

High‐mobility group protein 1 (HMGB1) is a ligand known to bind to the receptor for advanced glycation end products (RAGE), and it can activate nuclear factor‐κB (NF‐κB) to mediate cellular damage. RAGE and Parkinson's disease (PD) are closely associated, but it remains unclear whether the HMGB1/RAGE/NF‐κB signaling pathway contributes to the pathophysiology of PD.

**Methods:**

PD was induced by administration of 6‐hydroxydopamine (6‐OHDA), while RAGE was inhibited using an inhibitor, FPS‐ZM1. The grip strength test and Morris water maze were used to evaluate sensorimotor and memory skills. Then detect the expression levels of RAGE, HMGB1, and NF‐κB in the striatal sample using immunohistochemistry, western blotting, and RT‐qPCR.

**Results:**

(1) In PD rats, treatment with FPS‐ZM1 improved learning and memory ability and alleviated sensorimotor deficits. (2) The striatum of PD rats exhibited a significant increase in the number of HMGB1‐, RAGE‐, and NF‐κB‐positive cells, which could be reduced through the administration of FPS‐ZM1. Immunofluorescence double‐labeling results indicated that NeuN‐positive neurons were the primary sites of HMGB1‐, RAGE‐, and NF‐κB‐positive responses. Furthermore, these double‐labeled neurons demonstrated a significant increase following 6‐OHDA‐induced depletion of striatal dopamine (DA). However, the FPS‐ZM1 administration considerably attenuated these changes. (3) The treatment of FPS‐ZM1 significantly reduced the increase in protein expression of HMGB1, RAGE, and NF‐κB that followed striatal DA depletion. Similarly, *NF‐κB* and *RAGE* mRNA expression were increased by striatal DA deprivation; however, injection of FPS‐ZM1 significantly reduced these changes.

**Conclusion:**

The HMGB1/RAGE/NF‐κB signaling pathway plays a critical role in the pathogenesis of striatal neuronal damage in PD, highlighting its potential as a therapeutic target.

## Graphical abstract

In Parkinson's disease, the death of dopamine neurons in the substantia nigra, resulting in a decrease in dopamine levels in the striatum. This leads to striatal damage through the HMGB1/RAGE/NF‐κB signaling pathway, thereby inducing motor and cognitive dysfunction.

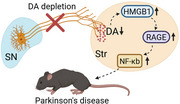

## 1 Introduction

Parkinson's disease (PD) is a neurological condition characterized by the death and degeneration of dopamine neurons in the substantia nigra of the midbrain, which results in a substantial decrease in dopamine levels in the striatum. (Dauer and Przedborski 2003) Despite numerous studies aimed at elucidating the pathophysiological mechanisms of PD, the exact causes remain unclear. Among the potential explanations for neurodegeneration in PD patients, neuronal death and chronic neuroinflammation have been implicated. (Hirsch and Hunot 2009) However, the evolution of intracerebral chronic inflammation and its specific impact on PD pathophysiology remain challenging to ascertain.

The RAGE expression is significantly upregulated in patients with Parkinson's disease, which has been linked to inflammation, oxidative stress, and protein aggregation in neurons, contributing to the pathophysiology of various diseases. According to mounting evidence, RAGE plays a critical role in triggering and maintaining inflammatory responses, which contribute to neurodegeneration. Therefore, targeting the RAGE signaling pathway may represent an effective strategy for treating Parkinson's disease. (Dalfo et al. 2005, Dong et al. 2022; Sathe et al. 2012; Bayarsaikhan et al. 2015; Jiang et al. 2018; Gomez and Ferrer 2010; Viana et al. 2016) As an immunoglobulin superfamily 35‐kilodalton transmembrane receptor, RAGE mediates inflammatory responses by interacting with different ligands, like β‐amyloid peptide (Aβ), advanced glycation end products (AGE), S100 family proteins, and HMGB1. (Xie et al. 2013; Neeper et al. 1992) HMGB1 is a low‐molecular‐weight, non‐histone chromatin protein that interacts with RAGE, thereby activating pro‐inflammatory pathways. (Klune et al. 2008) In a number of human illnesses, such as cancer, neurodegenerative diseases, and autoimmune diseases, HMGB1 plays a critical role. (Cui et al. 2023) Inhibition of HMGB1 expression and transportation has been shown to decrease 1‐methyl‐4‐phenyl‐1,2,4,5‐tetrahydropyridine (MPTP)‐induced deterioration of dopaminergic neurons while also reducing RAGE levels in midbrain tissues. (Santoro et al. 2016) In addition, RAGE initiates a cascade of cellular signaling events upon ligand binding, which includes the expression of pro‐inflammatory cytokines, the induction of inflammatory responses, and the activation of NF‐κB. (Lin et al. 2009) NF‐κB is a transcription factor that has a critical role in the neurodegenerative process. It regulates the expression of several important cytokines, such as interleukin‐1β (IL‐1β), interleukin‐6 (IL‐6), and tumor necrosis factor‐α (TNF‐α). (Rasheed et al. 2011; Bierhaus et al. 2006) Notably, 6‐OHDA induces RAGE activation and increases its levels in the rat substantia nigra, leading to downstream signaling pathway activation and further induces inflammatory responses. (Targeted inhibition of RAGE in substantia nigra of rats blocks 6‐OHDA–induced dopaminergic denervation—PMC 2024) However, the concrete mechanism by which the HMGB1/RAGE/NF‐κB signaling pathway contributes to striatal neuron injury in the 6‐OHDA‐induced PD rat model remains unclear.

To address these questions, this study utilized a 6‐OHDA‐induced DA depletion model of PD to investigate and compare the characteristic changes in the HMGB1/RAGE/NF‐κB axis and striatal neurons through behavioral studies, immunohistochemistry, and Western blotting. Additionally, we examined the effects of RAGE inhibition on neuroinflammation and cellular injury in the striatum following DA depletion, aiming to uncover potential underlying mechanisms.

## 2 Methods

### 2.1 Animal Grouping and Model Establishment

#### 2.1.1 Animal Grouping

Sprague‐Dawley (SD) rats (8 weeks, 200–250 g) were obtained from the Sun Yat‐sen University Center for Experimental Animals. The ethics committee of Jinan University approved all procedures for the care and handling of the rats [NO.20220313‐12]. The study adhered closely to the Guide for the Care and Use of Laboratory Animals (the US National Institutes of Health guide for the care and use of laboratory animals), was closely followed during the conduct of this experiment. 19 SD rats (5 per cage) had unrestricted access to food and water, as well as a temperature of 22 ± 0.5°C, with a relative humidity of 40%–70%, and weresubjected to a 12‐h light/dark cycle. The cages were cleaned and replaced weekly. All surgical procedures utilized sodium pentobarbital (50 mg/kg, i.p.) as an anesthetic to minimize suffering.

Four groups of 50 experimental rats were randomly assigned: (1) the control group (*n* = 10), without any treatment; (2) the PD model group (*n* = 10), which received an injection of 6‐OHDA solution into the MFB; (3) the RAGE inhibitor FPS‐ZM1 treatment group (*n* = 10; 6‐OHDA+FPS‐ZM1); and (4) the sham group (*n* = 20), which underwent the same procedures as the other groups but received 0.9% saline solution instead of 6‐OHDA or FPS‐ZM1. As a high‐affinity, effective, multimodal blocker of RAGE V domain‐mediated ligand binding (Ki = 25, 148, and 230 nM, respectively, against A40, HMGB1, and S100B, binding to sRAGE), FPS‐ZM1 (cat. no. 553030, Sigma) is a non‐toxic, blood‐brain‐barrier‐permeant tertiary amide substance used in diverse fields of research, including diabetes, neuroinflammation, atrial fibrosis, etc. (Sun et al. 2022; Tan et al. 2025; FPS‐ZM1 2021) characterizing low toxicity and high selectivity, FPS‐ZM1 have not effect on exercise performance, respiration, circulation, and metabolism; thus, based on overall consideration, we have not set up a group that only injects FPS‐ZM1 as another control. (Targeted inhibition of RAGE in substantia nigra of rats blocks 6‐OHDA–induced dopaminergic denervation—PMC 2024; JCI 2025) After establishing the PD model, the 6‐OHDA+FPS‐ZM1 group received daily intraperitoneal injections of FPS‐ZM1 (1 mg/kg/d, 2 mL) for 1 week. Four weeks after surgery, animals received intraperitoneal anesthesia (sodium pentobarbital, 50 mg/kg, i.p.), were perfused with 4% paraformaldehyde, and fixed or fresh tissues were collected for western blot and immunohistochemistry experiments.

#### 2.1.2 PD Model

The 6‐OHDA‐induced DA depletion method was used to establish a PD model. (Deumens et al. 2002) 6‐OHDA (catalog no. H116; Sigma) was dissolved in 0.9% saline to achieve a concentration of 2 µg/µL, with ascorbic acid added to reach a concentration of 0.01% to prevent oxidative degradation of 6‐OHDA. Each rat was injected with 8 µL of this solution into the right middle forebrain bundle (MFB). The injection locations were as follows: medial‑lateral (ML): −0.19 mm, anterior‑posterior (AP): −3.6 mm, and dorsal‐ventral (DV): −8.2 mm. Five‐minute intervals were used to provide two 4 µL intermittent injections and after being in place for an additional 15 min, the needle was gradually withdrawn. Apomorphine (0.25 mg/kg, APO; catalog no. 2073/50, Tocris) was administered subcutaneously to the rats over the three weeks following the development of 6‐OHDA lesions. Then, we counted the number of contralateral 360° rotations within a 30‐min period. For further analysis, only rats that completed more than 210 total rotations were included. (EPO 2024) Tyrosine hydroxylase (TH) immunohistochemical staining was utilized to determine whether the model worked. The following experiments were performed on rats whose ipsilateral striatal hemisphere had lost more than 95% of its TH fibers. (EPO 2024; Hefti et al. 1980; Yuan et al. 2005; Zheng et al. 2019) Rats given 6‐OHDA had significantly fewer TH‐immunoreactive cells or fibers in the striatum or substantia nigra on the opposite side. The successful construction of the 6‐OHDA‐lesioned model is shown in previously published articles. (Ma et al. 2014; Zhu et al. 2019) Due to data reuse avoidance, we will not repeat the presentation here, and the absence of these experiments in the present study is a limitation.

### 2.2 Behavioural Tests

#### 2.2.1 Grip Strength Test (GST)

We conducted the GST following the established procedures, 4 weeks post‐surgery. (Shear et al. 1998) First, we suspended a wire with a diameter of 2 mm and a length of 35 cm, positioned 50 cm above the ground. We then timed the duration for which the rats hung onto the wire to evaluate their grip strength. The test was carried out three times a day for a total of 5 days of testing. All animals took part in these tests (*n* = 10 per group). The experimental conditions for the rats were kept concealed from the observers.

#### 2.2.2 Morris Water Maze Task

This experiment was conducted 5 weeks after the 6‐OHDA injection. Initially, the rats underwent training in the Morris water maze twice daily for five consecutive days. On the final day of training, a probe test was administered. (Vorhees and Williams 2006) The target platform, which had a diameter of 10 cm, was positioned in a fixed location. Each experiment involved releasing the animals from four predetermined starting places (east, south, west, and north) and letting them swim for up to 2 min or until they reached the target platform. If a rat successfully found and climbed onto the platform, it remained on it for 30 s to help it remember the position. If a rat couldn't find the platform within 2 min, it was manually transferred there and given 30 s to stay. During the test, the TopScan behavioral analysis system (Clever Sys. Inc., USA) was used to record the latency.

### 2.3 Immunohistochemistry and Immunofluorescence

For immunohistochemistry, standardized laboratory protocols were followed when immunostaining was done. (Sathe et al. 2012) Prior to brain removal, 0.4% phenobarbital sodium (50 mg/kg, i.p.) was used to anesthetize all of the rats. Initially, we removed rats' brains. Additionally, rats (n = 4/group) were transcardially perfused with 500 mL of PBS (0.1 M) to flush the blood out of the body and then 500 mL of paraformaldehyde (4% in 0.1 M phosphate buffer, pH 7.4) during the 4 weeks following the 6‐OHDA lesions. Subsequently, after being taken out, the brain tissue samples were placed in 4% paraformaldehyde and stored at 4°C overnight. Using a vibratome, 30 µm thick brain slices were cut off. The sections were gathered roughly at the interaural plane levels (10.70 mm to 8.74 mm). Prior to blocking non‐specific binding sites with 3% BSA (cat. no. B2064; Sigma) at room temperature for 0.5 h, the sections underwent a 30 min pre‐treatment that included incubation with 0.1% Triton‐X 100 and 0.3% H_2_O_2_. The primary antibodies were mixed with buffer (0.5% BSA + 0.3% Triton in 5 mL of PB), including mouse anti‐TH (1:1000; Millipore Cat# MAB318, RRID:AB_2201528), and rabbit anti‐HMGB1 (1:1000; Abcam Cat# ab18256, RRID:AB_444360), rabbit anti‐RAGE (1:400; Abcam Cat# ab3611, RRID:AB_303947), rabbit anti‐NF‐κB (1:200; ServiceBio Cat# GB11997, RRID:AB_3083517). Then we incubated sections with primary antibodies at 4°C for 24 h. Following rinsing, the sections were treated with diluted anti‐rabbit IgG secondary antibody (1:200; Sigma‐Aldrich Cat# SAB3700848, RRID: AB3094541) and anti‐mouse IgG secondary antibody (1:200; Sigma‐Aldrich Cat# M4280, RRID: AB_260521) for 3 h at room temperature. Next the sections were incubated at room temperature for 2 h while being treated in homologous PAP complex (1:200; Sigma‐Aldrich Cat# P1291, RRID: AB_1079562). 3,3'‐diaminobenzidine (cat. no. 11718096001; Sigma), which is 0.05% in 0.1 M PBS, pH 7.4, was used in the peroxidase reaction, which was conducted for 1–2 min at room temperature. Following mounting on gelatin‐coated slides, the sections were dehydrated, rendered permeable using xylene, and then covered in neutral balsam.

For immunofluorescence: sections were made by the conventional double‐labeled immunofluorescence method. In this experiment, the primary antibodies were diluted in 0.1 M PBS (pH 7.4) and combined with 0.3% Triton X‐100 and 0.5% BSA. The following primary antibodies were used: mouse anti‐NeuN (1:800; Millipore Cat# MAB377, RRID: AB_2298772), rabbit anti‐HMGB1 (1:1000; Abcam Cat# ab18256, RRID: AB_444360), rabbit anti‐RAGE (1:400; Abcam Cat# ab3611, RRID: AB_303947), and rabbit anti‐NF‐κB (1:200; ServiceBio Cat# GB11997 (also GB11997‐100), RRID: AB_3083517). After the sections were incubated with the diluted primary antibodies for a whole night at 4°C, they were rinsed. For visualization using confocal microscopy, the sections were incubated with fluorescein‐conjugated anti‐rabbit (1:200; Thermo Fisher Scientific Cat# A32740, RRID: AB_2762824) or anti‐mouse (1:200; Thermo Fisher Scientific Cat# A‐11029, RRID: AB_2534088). Finally, the brain slices were mounted on a slide with the DAPI‐Fluoromount‐G aqueous mounting medium (Electron Microscopy Sciences Cat#17985‐50), and images were taken by a microscope (C2, Nikon).

### 2.4 Western Blotting

The animals (*n* = 3/group) received pentobarbital sodium (50 mg/kg, i.p.) 4 weeks after the 6‐OHDA lesions to induce a profound anesthesia. We extracted and lysed the striatal tissue using the RIPA buffer (Beyotime). Following SDS‐PAGE (10%) separation, the protein samples (30 µL) were deposited onto the polyvinylidene difluoride (PVDF) membranes (cat. no. IPVH00010; Millipore). The membranes were blocked for 2 h with 5% dry skim milk in Tris‐buffered saline‐Tween20 and then incubated with the primary antibodies, including rabbit anti‐HMGB1 (1:1000; Abcam Cat# ab18256, RRID: AB_444360), rabbit anti‐RAGE (1:1000; Abcam Cat# ab3611, RRID: AB_303947), rabbit anti‐NF‐κB (1:3000; Proteintech Cat# 66535‐1‐Ig, RRID: AB_2881898), and rabbit anti‐β‐actin (1:2000; Millipore Cat# ABT1485, RRID: AB_3094552) for an additional night at 4°C on a shaker. Subsequently, horseradish peroxidase (HRP)‐conjugated secondary antibodies, goat‐anti‐rabbit IgG (1:5000; Abcam Cat# ab6721, RRID: AB_955447) and goat‐anti‐mouse IgG (1:5,000; Abcam Cat# ab205719, RRID: AB_2755049), were applied to the membranes and incubated for 2 h at room temperature. The Bio‐Rad system (Bio‐Rad GelDoc XR+ system; Bio‐Rad Laboratories, Inc.) was used to visualize the protein bands, and the National Institutes of Health's ImageJ v1.8.0 program was used to quantify the data.

### 2.5 RT‐qPCR

After deep anesthesia with sodium pentobarbital (50 mg/kg, i.p.), the rats (*n* = 3 groups) were euthanized during the 4 weeks after the 6‐OHDA lesions. Then, each rat's striatum was taken out of the brain. Using the TRIzol reagent (Invitrogen; Thermo Fisher Scientific, Inc.), total RNA was extracted in accordance with the manufacturer's instructions. The SuperScript VILO cDNA Synthesis kit (cat. no. 11754250, Invitrogen; Thermo Fisher Scientific, Inc.) was applied to synthesize cDNA. In order to inactivate the reverse transcriptase, the samples were incubated on the PCR apparatus for 60 min at 42°C. On an ABI PRISM 7000 Sequence Detection (Applied Biosystems; Thermo Fisher Scientific, Inc.), qPCR was carried out using SYBR‐Green Master Mix (ABI; Thermo, Inc.). 45 cycles at 95°C for 30 s and 60°C for 30 s are then performed after five minutes at 50°C and 10 min at 95°C. The 2‐Cq technique was used to measure relative gene expression. (Livak and Schmittgen 2001) The following primers were utilized: *HMGB1* forward, 5′‐AAAGGAGATCCTAAGAAGCCGA‐3′; *HMGB1* reverse, 5′‐TCATAACGAGCCTTGTCAGCC‐3′; *RAGE* forward, 5′‐TGAGACGGGACTCTTCACGCT‐3′; *RAGE* reverse, 5′‐CACCTTCAGGCTCAACCAACA‐3′; *NF‐κB* forward, 5′‐GCTCCTTTTCTCAAGCCGATGT ‐3′; *NF‐κB* reverse, 5′‐CGTAGGTCCTTTTGCGTTTTTC‐3′; *β‐actin* forward, 5′‐GAACCCTAAGGCCAAC‐3′; and *β‐actin* reverse, 5′‐TGTCACGCACGATTTCC‐3′. The cycling conditions were those that had already been mentioned. (Zheng et al. 2019) The 7500 system SDS software v.2.0.6 (Applied BiosystemsTM; cat. no. 4377354; Thermo, Inc.) was used to analyze the melting curves.

### 2.6 Data Collection and Statistical Analysis

Slices from the interaural plane (8.74 mm–10.70 mm), according to the Paxinos and Watson atlas, were approximately obtained. The motor symptoms of Parkinson's disease are closely related to the dorsolateral striatum. Adjacent sections were immunostained for HMGB1, RAGE, and NF‐κB at each level of the striatum. The striatum's HMGB1, RAGE, and NF‐κB‐positive neurons were evenly dispersed throughout, according to LM observation. The researchers were blinded to the different experimental types. Using the previously mentioned techniques, the density of neurons was counted. In five randomly chosen squares with a side length of 100 µm, HMGB1‐, RAGE‐, and NF‐κB‐positive neurons were counted. Each rat in every group had five brain slices chosen for immunohistochemical staining and subsequent statistical analysis.

All data were statistically analyzed using SPSS 20.0 (RRID:SCR_016479, IBM). Experimental results are presented as means ± standard deviations (x̄ ± s). A one‐way analysis of variance (ANOVA) was employed to compare data across multiple groups, while an independent samples *t*‐test was utilized to compare data between two groups. The statistical significance among the three groups was assessed using the least significant difference post hoc tests. A *p*‐value of less than 0.05 indicates that the difference is statistically significant.

## 3 Results

Statistics revealed no significant differences between the PD model group and the sham group. Therefore, unless otherwise specified, only the control group, the 6‐OHDA group, and the 6‐OHDA+FPS‐ZM1 group are shown in the subsequent experiments.

### 3.1 RAGE Inhibitor FPS‐ZM1 Treatment Alleviated the Behavioral Impairments of PD Model Rats

**FIGURE 1:**
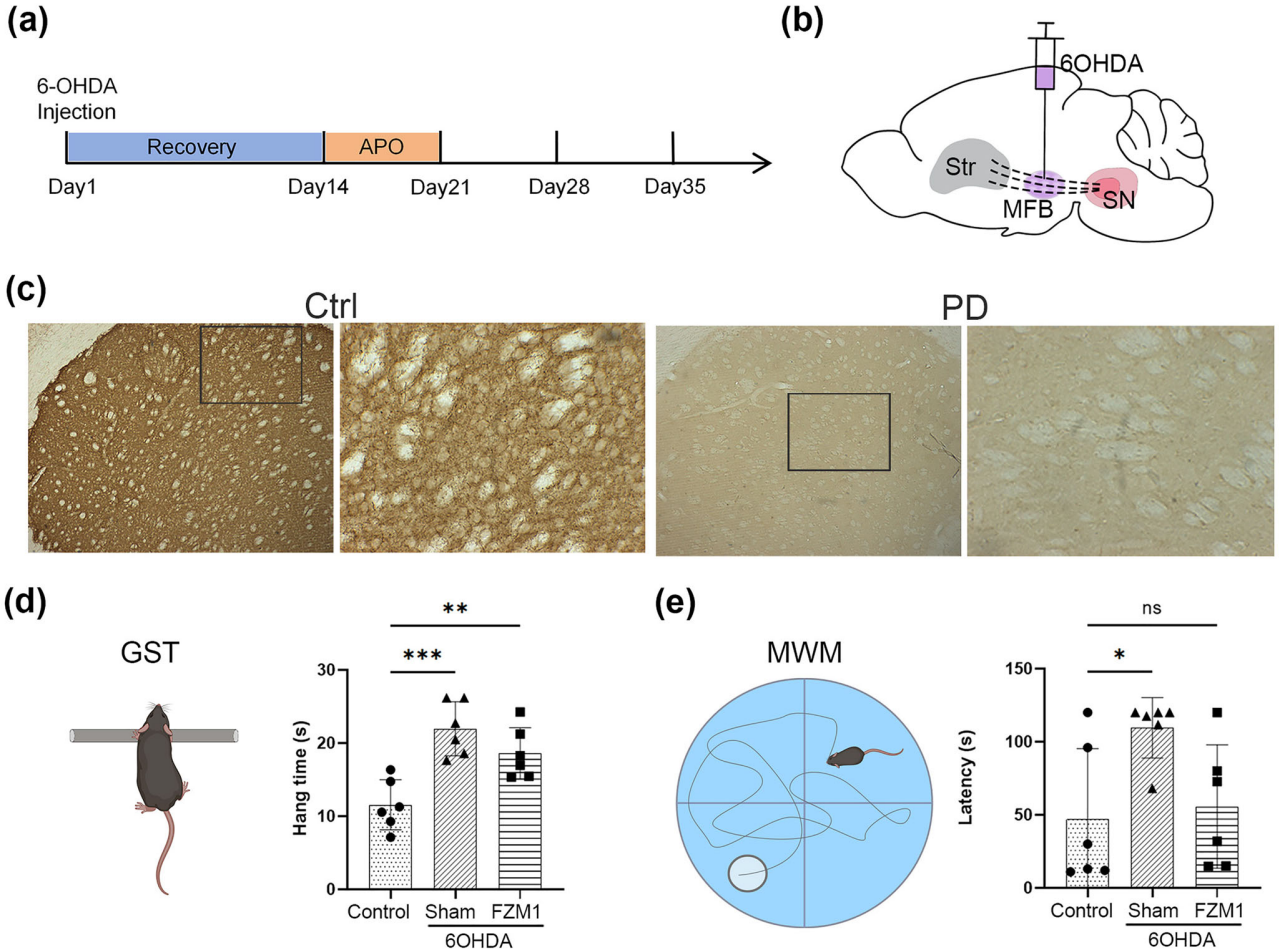
**Schematic plot and measurements obtained from the behavioural tests and their comparisons**. **(a)** Timeline of the experiment, **(b)** Schematic of intrastriatal 6‐OHDA injection, **(c)** TH staining for PD model group and the control group, **(d)** Grip test schematic and statistics, and **(e)** Morris water maze schematic and statistics. Values expressed as the group mean ± SD; one‐way ANOVA. ap < 0.05 vs. control; bp < 0.05 vs. 6‐OHDA group. **Abbreviations**: APO, Apomorphine; Ctrl, control; FPS‐ZM1, N‐benzyl‐4‐chloro‐N‐cyclohexylbenzamide; GST, Grip strength test; MFB, middle forebrain bundle; MWM, Morris water maze; SN, substantia nigra; Str, striatum; 6‐OHDA, 6‐hydroxydopamine. ***p < 0.001, **p < 0.01, and *p < 0.05.

Previous studies from our laboratory confirmed that 6‐OHDA‐induced DA depletion causes acute behavioral deficits in experimental rats. (Ma et al. 2014) We evaluated the mice's grip strength and their capacity for learning and memory following the procedure based on the timeline, and 6‐OHDA was accurately injected into the MFB (Figures 1a, b). The TH staining in the PD group showed a lighter color for negative results. While the control group showed a darker staining color for TH. The results of the GST showed that the PD model rats in the 6‐OHDA group (21.98 ± 1.51 s; shown in Figure 1c) were hanging for noticeably longer than the rats in the control group (11.56 ± 1.40 s, *P* < 0.05; shown in Figure 1d). Furthermore, a significant increase in latency time was observed between the 6‐OHDA group (109.5 ± 8.41 sec) and the control group (47.00 ± 19.75 s; *P* < 0.05; shown in Figure 1e) in the learning and memory evaluations conducted using the Morris water maze test. After administering the RAGE inhibitor FPS‐ZM1, the rats had a substantially shorter hanging time (18.60 ± 1.44 s; *P* < 0.05; shown in Figure 1d) than the animals in the 6‐OHDA group. Additionally, the latencies of the rats in the FPS‐ZM1 group (55.78 ± 17.23 s; *P* < 0.05; shown in Figure 1e) were shorter than the 6‐OHDA group rats.

### 3.2 FPS‐ZM1 Decreased HMGB1+ Neuron Density in Striatum

**FIGURE 2:**
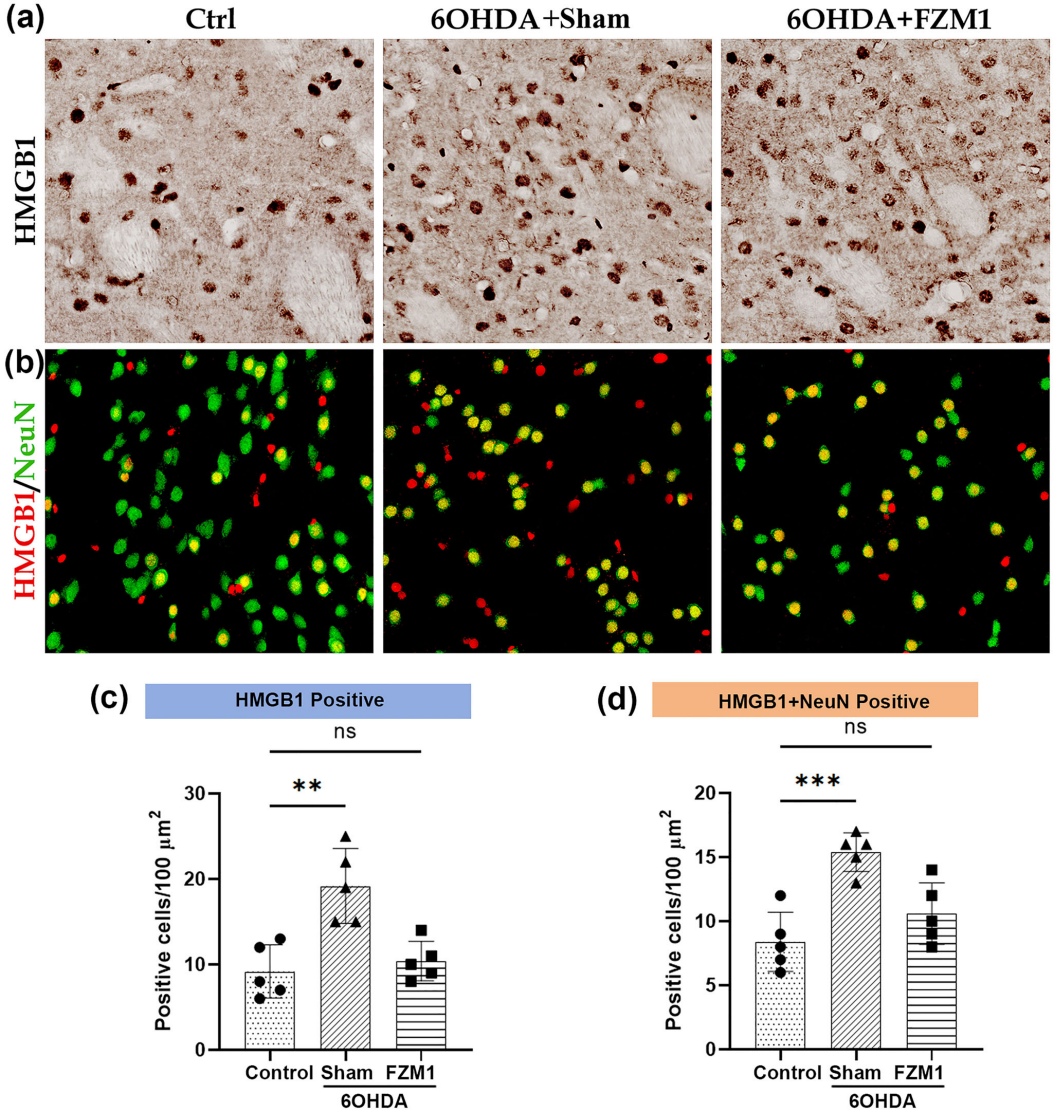
**FPS‐ZM1 decreased HMGB1 + neuron density in striatum**. **(a)** HMGB1 immunohistochemistry in the striatum of control, 6‐OHDA, and 6‐OHDA+FPS‐ZM1 groups, **(b)** Immunofluorescence showing NeuN (green) and HMGB1 (red). The 6‐OHDA group exhibited increased NeuN‐HMGB1 colocalization vs. control, which was reduced in the 6‐OHDA+FPS‐ZM1 group, **(c)** Mice injected with 6‐OHDA had significantly more HMGB1‐positive cells than those in the other two groups, and **(d)** HMGB1/NeuN double‐labeling showed a similar pattern. **Abbreviations**: Ctrl, control; FPS‐ZM1, N‐benzyl‐4‐chloro‐N‐cyclohexylbenzamide; 6‐OHDA, 6‐hydroxydopamine. ***p < 0.001, **p < 0.01, and *p < 0.05.

In both normal and pathological circumstances, HMGB1 plays a crucial regulatory role as a ligand for RAGE. The control group exhibited a more pronounced immunoreaction for HMGB1, with positive cells evenly distributed throughout the striatum, displaying a spherical granular appearance. The 6‐OHDA group demonstrated a significantly higher density of positive cells (19.20 ± 1.96) compared to the control group (9.20 ± 1.39; *p* < 0.05; Figures 2a, c). However, the 6‐OHDA + FPS‐ZM1 group showed a markedly lower density of HMGB1+ granules (10.40 ± 1.03) than the 6‐OHDA group (19.20 ± 1.96; *p* < 0.05; Figures 2a, c). The primary location of these HMGB1+ structures within the NeuN+ neuronal nuclei was verified by double‐labeling. The 6‐OHDA group exhibited a considerably higher number of double‐labeled cells (15.40 ± 0.68) compared to the control group (8.40 ± 1.03, *p* < 0.05) (Figures 2b, d). Additionally, there were 10.40 ± 0.93 fewer double‐labeled cells in the 6‐OHDA+FPS‐ZM1 group compared to the 6‐OHDA group (15.40 ± 0.68, *p* < 0.05, Figures 2b, d).

### 3.3 FPS‐ZM1 Partially Inhibits the Response of RAGE Induced by DA Depletion

**FIGURE 3:**
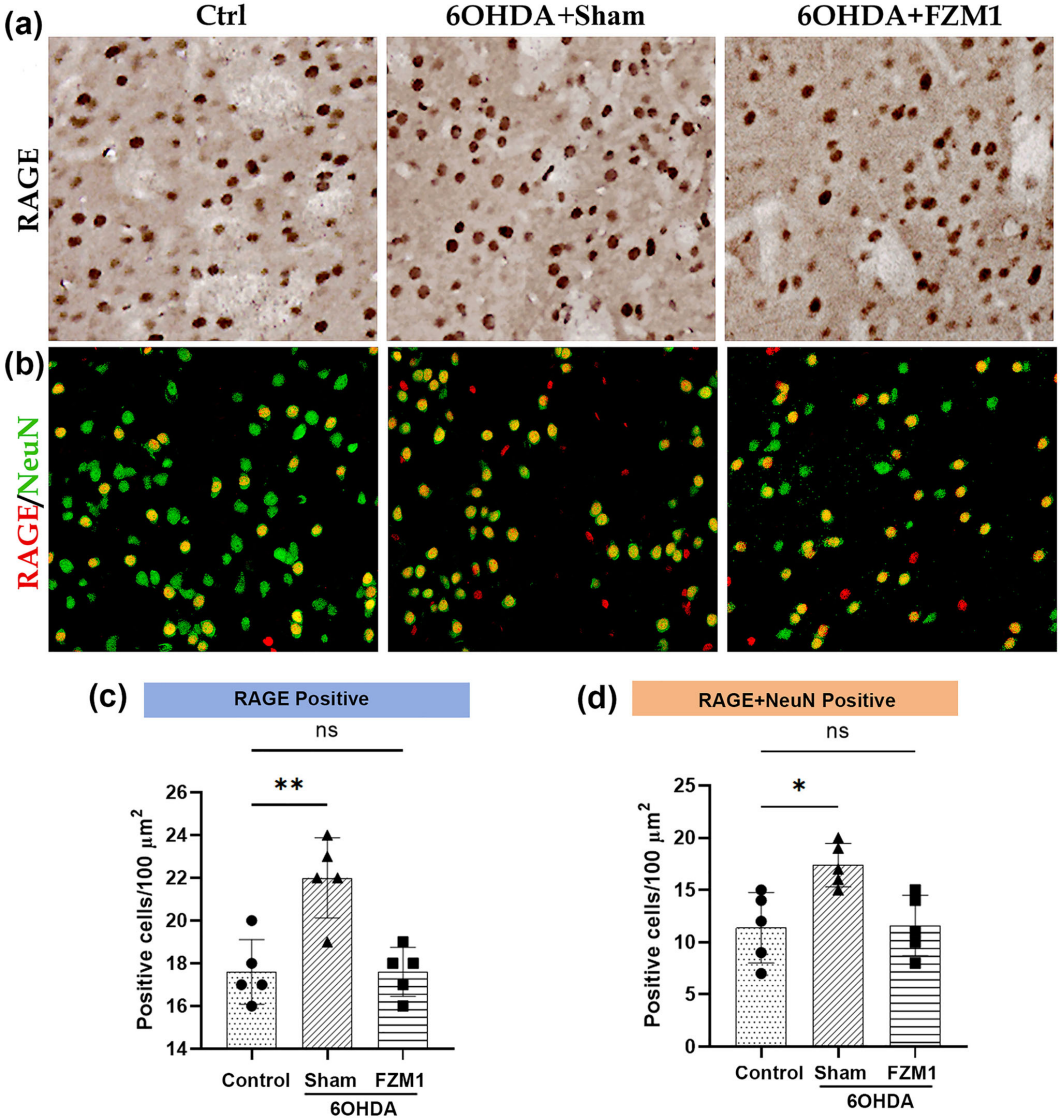
**FPS‐ZM1 partially inhibits the response of RAGE induced by DA depletion**. **(a)** RAGE immunohistochemistry in the striatum of control, 6‐OHDA, and 6‐OHDA+FPS‐ZM1 groups, **(b)** Immunofluorescence showing NeuN (green) and RAGE (red). Increased NeuN‐RAGE colocalization was observed in the 6‐OHDA group compared to control, which was reduced in the 6‐OHDA+FPS‐ZM1 group, **(c)** The 6‐OHDA group showed significantly more RAGE‐positive cells than the other groups, and **(d)** Double‐labeling of RAGE/NeuN group has same changing trends. **Abbreviations**: Ctrl, control; FPS‐ZM1, N‐benzyl‐4‐chloro‐N‐cyclohexylbenzamide; 6‐OHDA, 6‐hydroxydopamine. ***p < 0.001, **p < 0.01, and *p < 0.05.

The striatum of normal rats exhibited a notable number of uniformly dispersed RAGE+ cells (17.60 ± 0.68), according to the investigation. However, this number was not as high as that observed in the 6‐OHDA group (Figures 3a, c; 22.00 ± 0.84, *P* < 0.05). In contrast, the 6‐OHDA+FPS‐ZM1 group displayed a considerably lower number of RAGE‐positive cells (17.60 ± 0.51, *P* < 0.05; Figures 3a, c). Immunofluorescence double‐labeling was employed to confirm the colocalization of RAGE and NeuN. The results indicated that there were significantly more double‐labeled cells (17.40 ± 0.93) in the 6‐OHDA group than in the control group (11.40 ± 1.50, *P* < 0.05). Furthermore, after FPS‐ZM1 was administered, there was a substantial decrease in the number of double‐labelled cells (11.60 ± 1.29, P<0.05; Figures 3b, d) relative to the 6‐OHDA group.

### 3.4 Inhibit RAGE Decreased NF‐κB Expression in the DA Depleted Striatum

**FIGURE 4:**
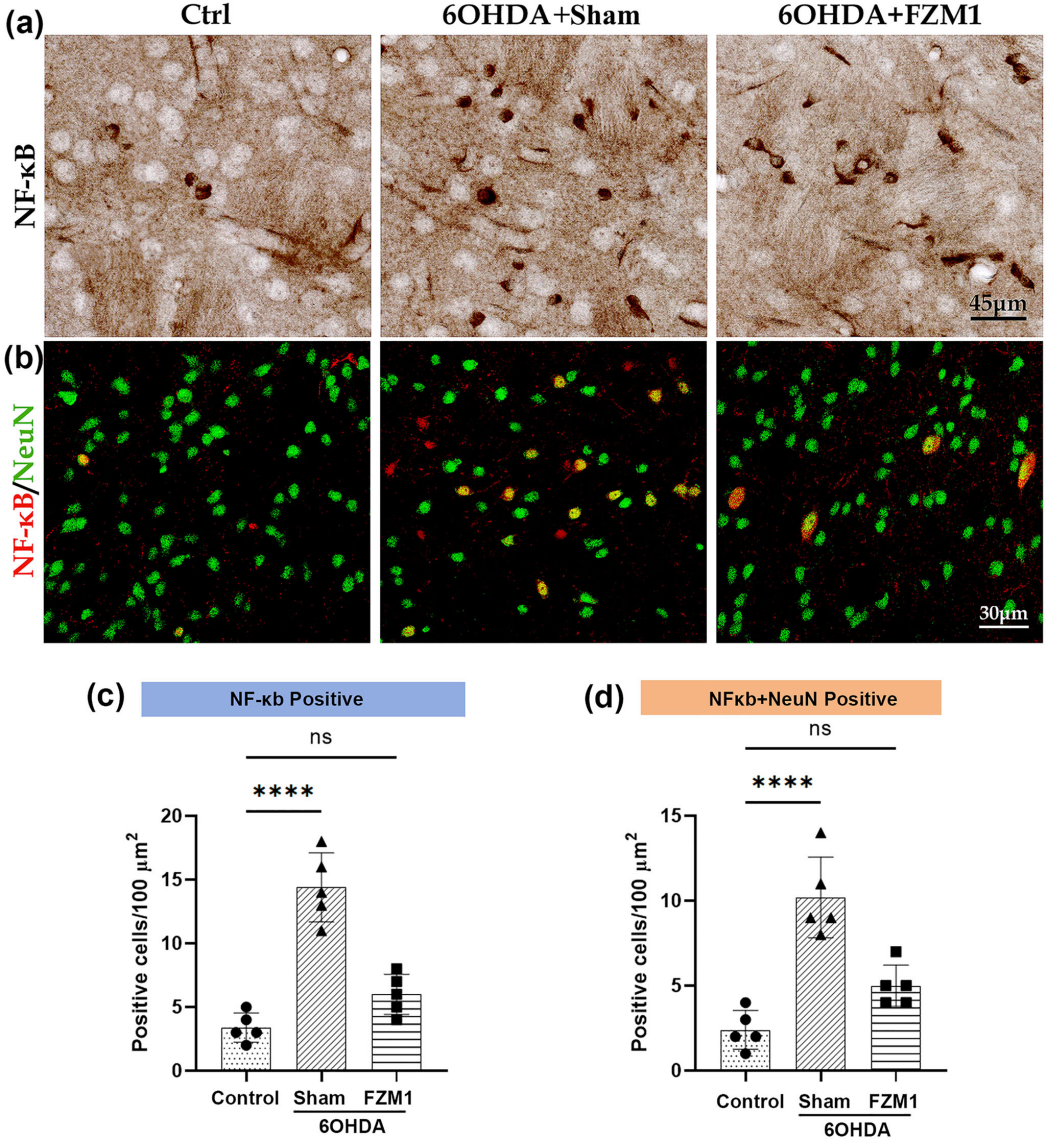
**Inhibiting RAGE decreased NF‐κB expression in the DA‐depleted striatum**. **(a)** NF‐κB immunohistochemistry in the striatum of control, 6‐OHDA, and 6‐OHDA+FPS‐ZM1 groups, **(b)** Increased NeuN–NF‐κB colocalization was observed in the 6‐OHDA group compared to control, which was reduced in the 6‐OHDA+FPS‐ZM1 group, **(c)** The 6‐OHDA group had significantly more NF‐κB‑positive cells than the other groups, and **(d)** Double‐labeling of HMGB1/NeuN group has the same changing trends. **Abbreviations**: Ctrl, control; FPS‐ZM1, N‐benzyl‐4‐chloro‐N‐cyclohexylbenzamide; 6‐OHDA, 6‐hydroxydopamine. ***p < 0.001, **p < 0.01, and *p < 0.05.

Molecular biology studies have confirmed that RAGE is an upstream protein of NF‐κB and regulates the protein levels and functions of NF‐κB through various protein kinases. In the striatum of the control group (3.40 ± 0.71), immunohistochemical staining only revealed a few NF‐κB‐positive cells, whereas 6‐OHDA‐induced striatal DA depletion dramatically increased the number of NF‐κB‐positive cells (14.65 ± 0.68; *P* < 0.05; Figures 4a, c) in comparison to the control group. After treatment with FPS‐ZM1, the number of NF‐κB‐positive cells (6.43 ± 0.55; *p* < 0.05; Figures 4a, c) significantly decreased compared to the 6‐OHDA group. Immunofluorescence double‐labeling revealed the colocalization of NF‐κB and NeuN, with the 6‐OHDA group (10.50 ± 0.37) having significantly more double‐labeled cells than the control group (2.47 ± 0.25; *p* < 0.05; Figures 4b, d). Approximately 20% of NF‐κB‐positive cells in the 6‐OHDA group did not display double‐labelling with NeuN. In comparison to the 6‐OHDA group, the 6‐OHDA+FPS‐ZM1 group showed significantly fewer double‐labelled cells (5.15 ± 0.32; *p* < 0.05; Figures 4b, d).

### 3.5 FPS‐ZM1 Downregulated Striatal HMGB1, RAGE, NF‐κB Protein, and mRNA Levels Induced by DA Depletion

**FIGURE 5:**
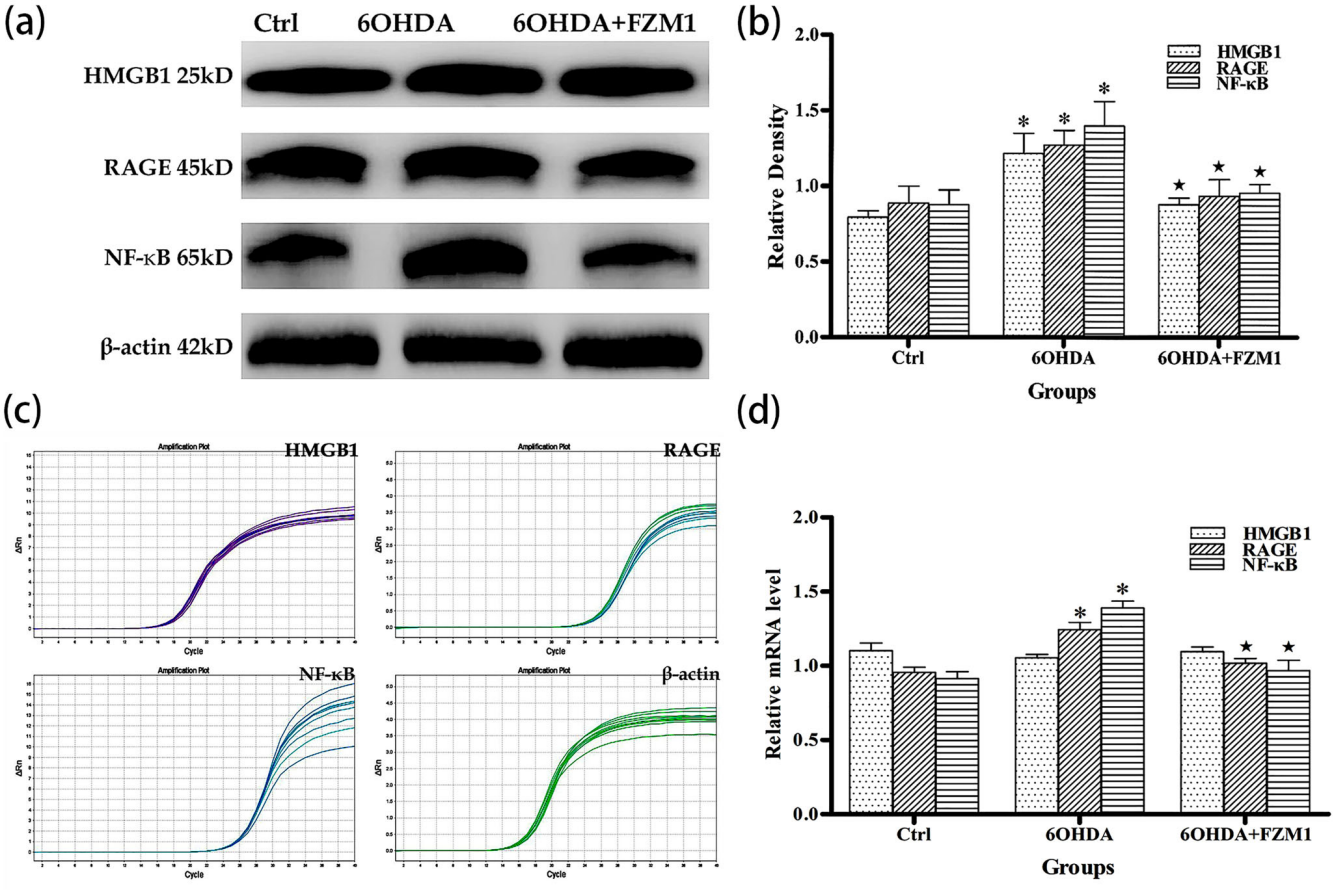
**The proteins and mRNA expression levels of the HMGB1, RAGE, and NF‐κB**. **(a)** Western blotting, **(b)** Quantification of HMGB1, RAGE, and NF‐κB protein expression. Levels of all three proteins were higher in the 6‐OHDA group than in controls, but lower in the 6‐OHDA+FPS‐ZM1 group, **(c)** Amplification curves of *HMGB1*, *RAGE*, *NF‐κB*, and *actin* mRNA, and **(d)** mRNA quantification in striatal tissue. *RAGE* and *NF‐κB* mRNA levels were significantly increased in the 6‐OHDA group compared to controls and decreased in the 6‐OHDA+FPS‐ZM1 group. *HMGB1* mRNA expression showed no significant differences among groups. Data were analyzed by one‐way ANOVA and expressed as the means ± SD (*n* = 3/group). *p < 0.05, vs. each control group; ★*p* < 0.05, vs. each 6‐OHDA group. **Abbreviations**: Ctrl, control; FPS‐ZM1, N‐benzyl‐4‐chloro‐N‐cyclohexylbenzamide; 6‐OHDA, 6‐hydroxydopamine.

Based on the immunohistochemical detection described above, Western blotting was employed to further investigate the changes in HMGB1, RAGE, and NF‐κB protein levels induced by striatal DA depletion, as well as the effect of FPS‐ZM1 on these changes. In the control striatum, HMGB1 (0.79 ± 0.04), RAGE (0.88 ± 0.11), and NF‐κB (0.88 ± 0.10) were expressed in the control striatum. Striatal levels of HMGB1 (1.22 ± 0.13; *p* < 0.05), RAGE (1.27 ± 0.10; *p* < 0.05), and NF‐κB (1.40 ± 0.16; *p* < 0.05) were significantly increased following 6‐OHDA‐induced DA depletion. Following DA depletion, the injection of FPS‐ZM1 significantly reduced the levels of HMGB1 (0.88 ± 0.04; *p* < 0.05), RAGE (0.93 ± 0.11; *p* < 0.05), and NF‐κB (0.95 ± 0.06; *p* < 0.05) proteins (Figures 5a, b).

RT‐qPCR was utilized to explore the alterations in *HMGB1*, *RAGE*, and *NF‐κB* mRNA levels induced by striatal DA depletion and the impact of FPS‐ZM1 on these changes. The results revealed that the HMGB1 expression levels in the control group (1.102 ± 0.05) did not differ significantly from those in the 6‐OHDA group (1.054 ± 0.02) or the 6‐OHDA+FPS‐ZM1 group (1.096 ± 0.03; *p* > 0.05; Figures 5c, d). However, RT‐qPCR analyses indicated that RAGE and NF‐κB expression levels were up‐regulated in the 6‐OHDA group (1.24 ± 0.05; 1.39 ± 0.05, respectively) in comparison to the control group (0.96 ± 0.03; 0.91 ± 0.05; *p* < 0.05; Figure 5). Similarly, RAGE and NF‐κB expression levels were lower in the 6‐OHDA+FPS‐ZM1 group (1.02 ± 0.03; 0.97 ± 0.07) compared to the 6‐OHDA group (1.24 ± 0.05; 1.39 ± 0.05; *p* < 0.05; Figures 5c, d).

## 4 Discussion

We utilized a rat model of 6‐OHDA‐induced nigrostriatal degeneration to investigate whether inhibiting RAGE activity could improve motor coordination and mitigate injury in dorsolateral striatal neurons. Following 6‐OHDA‐induced DA depletion, the dorsolateral striatum had considerably more RAGE‐positive cells and elevated levels of *RAGE* mRNA expression compared to the control group, suggesting RAGE's involvement in the development and progression of PD. However, rats in the 6‐OHDA+FPS‐ZM1 group, which were treated with the RAGE inhibitor FPS‐ZM1, exhibited improved motor coordination and preserved striatal neuron function compared to rats in the 6‐OHDA group. The findings from earlier tests were confirmed by Western blot analysis, which revealed that the levels of HMGB1, RAGE, and NF‐κB proteins in the 6‐OHDA‐induced PD group were considerably greater than those in both the control group and the FPS‐ZM1 treatment group. Therefore, this study confirms that RAGE likely plays a critical role in the injury of striatal neurons and the pathogenesis of PD through specific mechanisms.

The pathogenesis of PD involves various potential reactions of neuronal cells, including inflammation, apoptosis, and autophagy; nevertheless, the exact mechanisms remain unclear. It has been established that RAGE is a key player in the degenerative process of PD. RAGE interacts with numerous ligands to mediate a variety of biological activities in distinct cell types, such as pro‐inflammatory pathways, immune responses, oxidative stress, and cell migration. (Gao et al. 2014; Batkulwar et al. 2015; Rinaldi et al. 2019; Teismann et al. 2012; Tóbon‐Velasco et al. 2014) The abnormal increase in RAGE expression and activation, along with subsequent immune responses, is assumed to have a role in the pathogenic processes of several human illnesses, including neurodegenerative diseases like PD. Clinical studies have reported significantly higher levels of RAGE in the frontal cortex of PD patients in contrast to healthy individuals. (Dalfo et al. 2005) In the MPTP‐induced PD model in mice, the loss of RAGE effectively attenuated MPTP‐induced cytotoxicity and reduced RAGE‐mediated inflammatory responses, thereby delaying the start of PD. (Teismann et al. 2012)

RAGE interacts with many ligands and is important during the degenerative stage of Parkinson's disease. A key RAGE ligand is HMGB1. (Klune et al. 2008) The dorsolateral striatum of the 6‐OHDA group in the current experiment showed considerably more HMGB1‐positive cells and HMGB1 protein compared to the normal control group, demonstrating HMGB1's involvement in the pathological process of 6‐OHDA‐induced nigrostriatal degeneration. Precedential experiments have shown that the neurotoxicity of 6‐OHDA is decreased when monoclonal antibodies are administered to PD model rats to inhibit HMGB1. (Sasaki et al. 2016) Previous literature has established a connection between RAGE and the association of HMGB1 with several neurodegenerative diseases. (Rong et al. 2005) In addition, compared to the 6‐OHDA group, the dorsolateral striatum exhibited a reduced number of HMGB1‐positive cells and lower levels of HMGB1 protein, attributed to decreased RAGE activity. Histones, which are typically found in the nucleus alongside HMGB1, can be released into the cytoplasm under specific pathological conditions, contributing to various disease processes. (Paudel et al. 2018) Previous research has shown that HMGB1 transfers from the nucleus to the cytoplasm in the MPTP‐induced Parkinsonian animal model and in the cell cycle arrest caused by the widely used PD model medication, rotenone. We will confirm whether this phenomenon occurs in the 6‐OHDA‐induced PD model of SD rats in further research. (Dutta et al. 2025; Kim et al. 2019) Hence, its possibility exists that although HMGB1's mRNA level remained unchanged, the protein level was mainly moved out of the nucleus and into the cytoplasm and extracellular space. This suggests that HMGB1 and RAGE interaction may be involved in the pathophysiology of PD brought on by 6‐OHDA.

The binding of RAGE to its ligand HMGB1 induces intracellular signal transduction. Based on the results of the present study, NF‐κB expression was found to be elevated in striatal neurons following 6‐OHDA‐induced DA depletion. RAGE activation triggers rapid production of reactive oxygen species (ROS) and enhances the activity of inflammatory pathways through RAGE signal transduction‐dependent mechanisms, ultimately resulting in NF‐κB activation. (Villarreal et al. 2011; Rouhiainen et al. 2013) NF‐κB regulates the expression of various important cytokines, including TNF‐α, IL‐1β, and IL‐6. These cytokines take part in the development of neurodegeneration. (Rasheed et al. 2011; Bierhaus et al. 2006) Furthermore, these soluble cytokines and their receptors not only draw in and activate glial cells, but they also trigger signalling pathways that result in the production of several pro‐inflammatory mediators in a positive feedback loop. (Bierhaus et al. 2006) However, the exact molecular mechanisms driving the establishment and maintenance of the pro‐inflammatory microenvironment and subsequent cell injury are still not fully understood. In this study, NF‐κB expression in rat striatal neurons was downregulated following the inhibition of RAGE activity in rats with 6‐OHDA‐induced DA depletion. This suggests that NF‐κB is involved in the mechanism by which RAGE and 6‐OHDA‐induced DA depletion contribute to neuronal injury. Recent reports indicate that RAGE promotes cellular injury by regulating autophagy, inflammation, and apoptosis. Nevertheless, certain limitations should be acknowledged.

However, we did not include a control group treated with FPS‐ZM1 alone. While this limits our ability to definitively rule out any baseline effects of the inhibitor itself, it is important to note that FPS‐ZM1 has been extensively characterized in previous studies as having no significant off‐target effects or toxicity at the dosage used and does not affect normal motor or cognitive performance in rats; even the administration of a 500‐fold therapeutic dose does not cause any significant toxicity. (JCI 2025) Furthermore, regarding HMGB1, while our mRNA data showed no change, the significant increase in protein levels and immunohistochemical signal strongly suggests post‐translational regulation and translocation, a phenomenon well‐documented in other PD models. The specific mechanisms involved here are the direction we are currently exploring. Consequently, following ligand interaction, the RAGE‐activated intracellular machinery leads to the dissociation of the p65 and p50 subunits of the NF‐κB complex from the inhibitory inhibitor of NF‐κB (IκB) subunit in the cytoplasm, resulting in the nuclear translocation of p65. In the future, we will put emphasis on research investigating the effect of RAGE inhibition on p65 and IκB is of great significance.

## 5 Conclusion

This study investigated and compared the effect of RAGE inhibition on HMGB1, RAGE, and NF‐κB in a PD rat model after. The findings suggest that, in addition to the traditionally recognized midbrain substantia nigra, understanding the injury mechanism and protection of striatal neurons targeting the HMGB1/RAGE/NF‐κB pathway could provide new targets for fundamental research and clinical treatment of PD.

## Author Contributions

The experiments were created and planned by W. L. L., X. F. Z., and Y. F. Z. The experiments were carried out by Y. F. Z., Z. A. D., L. P. S., and Z. Y. H., who also wrote the manuscript. Z. A. D., L. P. S., and T. C. handled data collection. The analysis of the data was done by L. J. J. and T. C. Participating in the manuscript's revision were Z. Y. H. and L. J. J. The final manuscript was read and approved by all authors.

## Funding

This work was funded by National Natural Science Foundation of China (82260264, 32200832, 81471288); Hunan Provincial Natural Science Foundation of China (2021JJ30561); Guangdong Basic and Applied Basic Research Foundation (2022A1515012553, 2023A1515111157).

## Ethics Statement

This study protocol was reviewed and approved by ethics committee of Jinan University, approval number [NO.20220313‐12].

## Conflicts of Interest

The authors declare no conflicts of interest.

## Data Availability

The data that support the findings of this study are available from the corresponding author upon reasonable request.
